# Critical Analysis and Quality Assessment of Nanomedicines and Nanocarriers in Clinical Trials: Three Years of Activity at the Clinical Trials Office

**DOI:** 10.3390/pharmaceutics14071438

**Published:** 2022-07-09

**Authors:** Diego Alejandro Dri, Elisa Gaucci, Ilaria Torrieri, Maria Carafa, Carlotta Marianecci, Donatella Gramaglia

**Affiliations:** 1 Clinical Trials Office, Italian Medicines Agency (AIFA), Via del Tritone 181, 00187 Rome, Italy; e.gaucci@aifa.gov.it (E.G.); d.gramaglia@aifa.gov.it (D.G.); 2 Department Drug Chemistry and Technologies (DCTF), Sapienza, University of Rome, Piazzale Aldo Moro 5, 00185 Rome, Italy; torrieri.1748856@studenti.uniroma1.it (I.T.); maria.carafa@uniroma1.it (M.C.)

**Keywords:** clinical trials, investigational medicinal products, nanocarrier, nanomedicine, quality, regulatory

## Abstract

Investigational medicinal products submitted over the course of 3 years and authorized at the Clinical Trials Office of the Italian Medicines Agency as part of a request for authorization of clinical trials were scrutinized to identify those encompassing nanomedicines. The quality assessment reports performed on the documentation submitted were analyzed, classifying and discussing the most frequently detected issues. The identification of nanomedicines retrieved and the information on their quality profiles are shared to increase the transparency and availability of information, providing feedback that can support sponsors in optimizing the quality part of the documentation and of the information submitted. Results confirm that nanomedicines tested as investigational medicinal products in clinical trials are developed and authorized in agreement with the highest standards of quality, meeting safety profiles according to the strong regulatory requirements in the European Union. Some key points are highlighted and indicate that the regulatory approach to innovation in a clinical trial setting could potentially be renewed to ride the wave of innovation, particularly in the nanotechnology field, capitalizing on lessons learned and still ensuring a strong and effective framework.

## 1. Introduction

Drug delivery systems can usually be categorized into organic nanostructures, mainly used in clinical treatment, or inorganic nanostructures, which find their application particularly in the diagnostic field. Depending on the active molecule to enclose, on the desired formulation and administration route, on the safety profile, and on the therapeutic rather than the diagnostic purpose, a different approach to the synthesis and selection of starting materials may apply [1,2]. Reviews and publications show how nanomedicines and nanocarrier-based delivery systems for drugs have future prospects in both therapeutic and diagnostic fields [3]. Nanomedicines, nanocarriers, and drug delivery systems are definitely an emerging and promising field of innovation in healthcare [4,5]. The excellent results achieved, with an increasing number of formulations reaching the market in the preceding decades [6,7], ultimately demonstrate that some of the challenges that this field of innovation is facing can be overcome when there is a common intent among the various stakeholders. In the case of the COVID-19 pandemic, the confluence of know-how, technological, economic, and regulatory efforts has supported the authorization of nanovaccines by regulatory bodies worldwide [8,9], even if in an emergency situation. However, there are still challenges ahead [10], and any further development in the field may suffer from the unavailability of dedicated regulatory guidelines or from non-standardized approaches to innovation. It is therefore crucial to identify those difficulties that hinder the clinical translation of nanomedicines and encourage high-quality and value, potentially low-cost, fast approachable nanotechnology innovation to address unmet medical needs [11].

**Figure 1:**
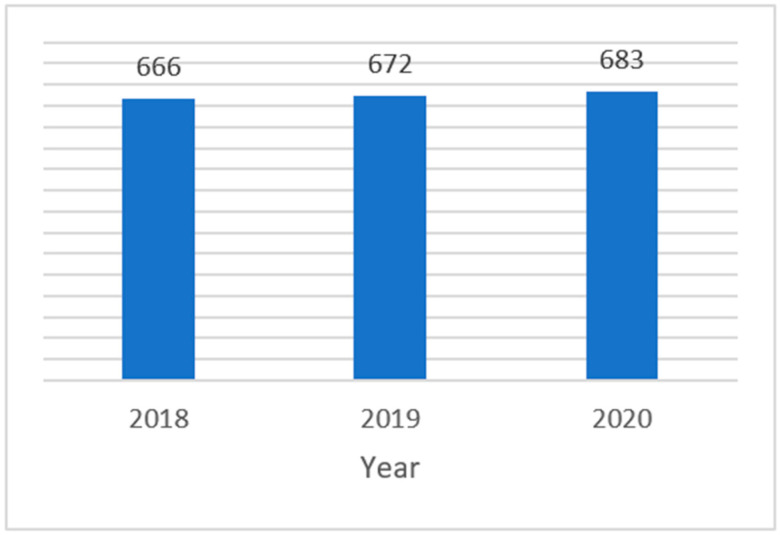
Number of CTs authorized per year at the CTO from 2018 to 2020.

There are publications and reviews illustrating how challenging is the translation from bench to the clinic [12,13,14]; however, there is limited information on the actual clinical application of innovation in the nanotechnology field when it comes to nanomedicines in clinical trials (CTs). It is our intent to provide valuable information to nanomedicines’ developers and sponsors of CTs to help identify some of the potential areas of difficulty, with considerations in terms of good manufacturing practice (GMP) and chemistry, manufacturing, and controls (CMC). A medicinal product can be authorized in the European Union (EU) only after its efficacy and safety are investigated in CTs [15,16,17,18,19]; the assessors of the national competent authorities (NCAs) ensure a positive benefit–risk profile assessing the protocols and the investigational medicinal products (IMPs). In this article, we retrieve, critically analyze, and discuss the quality documentation and data provided by the sponsors in the clinical trial applications (CTA) with a nanomedicine, submitted to the Clinical Trials Office (CTO) of the Italian Medicines Agency (AIFA) and authorized from 2018 to 2020. The total number of CTs authorized during the 3 years of this research is 2021, slightly increasing each year during the period, as shown in Figure 1.

Applications submitted through the Osservatorio Nazionale delle Sperimentazioni Cliniche (OsSC) [20], a national system, are assessed from different points of view, including regulatory, administrative, non-clinical, clinical, statistical, and of course quality perspective. The safety profile of IMPs can be confirmed only after the critical quality attributes are controlled and a suitable characterization is made available. Most of the information analyzed is commercially confidential, therefore data cannot be fully disclosed; however, results provided as aggregated data represent useful information, particularly for sponsors, and increase transparency for all stakeholders, triggering also regulatory reflections and highlighting critical key points to be further elaborated by the entire network.

## 2. Materials and Methods

Interventional CTs submitted and authorized from 2018 to 2020 at the CTO in AIFA were scrutinized to retrieve those involving IMPs that may be classified as nanomedicines. Structured data in the OsSC national system do not contain information on the presence of a nanotherapeutic tested as an IMP in a CT. Therefore, a manual process had to be implemented to assess, for every CT application, if the CMC quality documentation available in the Investigational Medicinal Product Dossier (IMPD) mentioned nanomedicine- or nanocarrier-related terms or if it contained information, descriptions, and analytical data suggesting the presence of a nanostructure, taking into consideration the terms of the JRC technical report [21].

The information managed is commercially confidential. Therefore, full open data cannot be disclosed. The number of CTs submitted every year to the CTO and authorized after the assessment is available through the national report on CTs in Italy [22]. For the purpose of our analysis, the list of CTs authorized in the period 2018–2020 has been further narrowed down to only those CTs testing an IMP without a marketing authorization, because we wanted to retrieve and analyze those new nanotherapeutics that had not yet received a marketing authorization and for whom complete information was not available. However, we included those IMPs declared in the CTA form as not having a marketing authorization because they were investigated with a different indication or formulation from the one already authorized. Reference products tested as comparators, placebos, and Phase IV CTs were also excluded. For a given CT, we did not retrieve more than one IMP involving a nanomedicine and a nanocarrier tested in the same study and we did not find that in the same CT any IMP was tested multiple times because of multiple pharmaceutical forms or strengths involved. We are reporting in Table 1 the number of CTs submitted between 2018 and 2020, the number of CTs authorized by the CTO, and the number of CTs within the scope of this research. During 2020, the first year of the COVID-19 pandemic, the number of IMPs without a marketing authorization tested in a CT dropped dramatically, in favor of testing-authorized medicinal products with a repurposing scope.

We analyzed the quality documentation available in the CMC section of the IMPDs and the quality-assessment outcome for all those 1241 CTs in scope according to the information declared by the sponsors in the CTA form. For the purpose of identifying the overall number of issues raised in the context of those CTs involving a nanomedicine as an IMP, we included in the list a few IMPs as duplicated when they were tested multiple times in different CTs, considering that a separate quality assessment was indeed performed for each CT.

After the quality assessment is completed by the assessor at the CTO, should any issue be identified during the assessment process, requests for clarification, additional data, or information are sent to the sponsors as grounds for non-acceptance. The sponsor has then the possibility to review the issues raised and to reply to the NCA. Only if the responses are considered acceptable, a final positive conclusion on the quality part is adopted, complementing the conclusion on the other parts of the dossier (regulatory, statistical, clinical, non-clinical) and contributing to the definition of a benefit–risk profile for the CTs and, in the end, to set a final decision on the application.

## 3. Results

**Figure 2:**
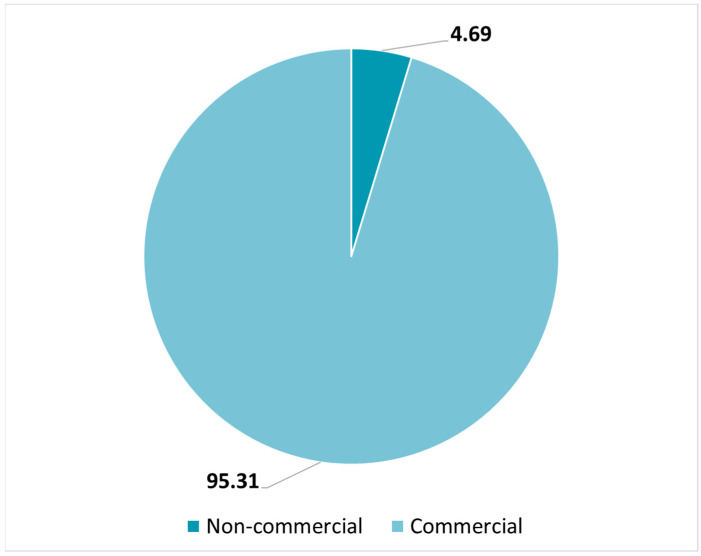
Percentage of commercial and non-commercial CTs assessed and authorized from 2018 to 2020, including a nanomedicine tested as an IMP.

In all, 22 IMPs that may be classified into categories attributed to nanomedicines were identified in CTs authorized in 2018 as a result of previously conducted research [23]. We are hereby capitalizing on those data, adding the CTs authorized during the following 2 years, 2019 and 2020, to the 2018 database. A total of 23 IMPs were further identified among CTs authorized in 2019, and the list is reported in Table A1. Among those, four contained an IMP already detected the previous year (N7 and 3 × N17) and three contained the same IMP (N28) but were included in the analysis because the IMP was evaluated in the context of a new CT. Instead, 19 IMPs were identified among CTs authorized in 2020 and the list is reported in Table A2. Among those, one contained an IMP already detected in 2018 and 2019 (N7), one already detected in 2019 (N34), and two detected twice in the same year (N42 and N53). As said above, they were included in the analysis. A total of 64 (3.17%) out of 2021 authorized CTs during the 3 years (2018–2020) included IMPs that may be classified as nanomedicines. Only 3 (4.69%) out of the 64 CTs were declared not to have a commercial nature. The vast majority (95.31%) were instead declared to be commercial, as reported in Figure 2.

### 3.1. Type of Nanomedicines

We are reporting all the IMPs that may be classified as nanomedicines, both those with a confirmed dimension in the nano-range and also those, the majority, without confirmation of the dimension but detected as having description, characteristics, or critical parameters that make them fit into one potential type of nanomedicine, as already reported for the analysis of the 2018 database.

**Figure 3:**
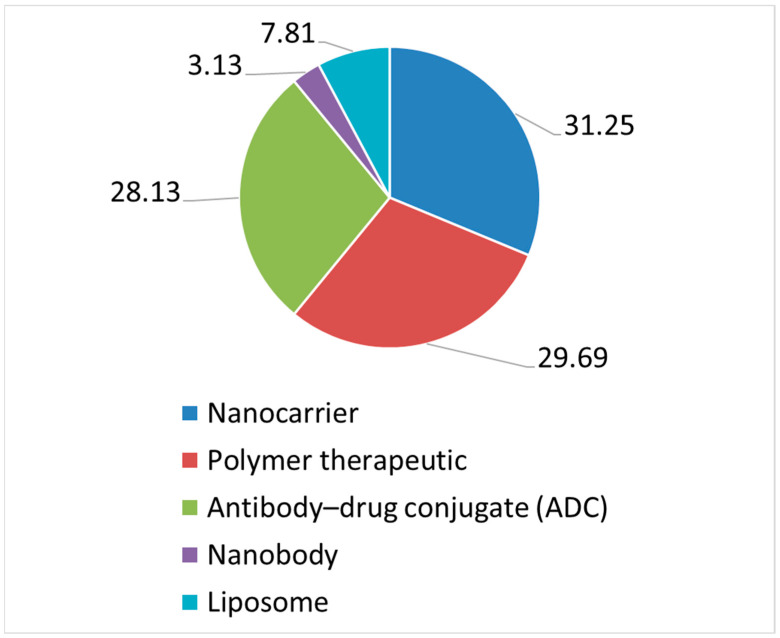
Categories attributable to nanomedicines identified in CTs assessed and authorized from 2018 to 2020.

IMPs were not declared as nanomedicines in the CTA form [24] nor in the IMPD; however, we classified them into a few types: nanocarriers (viral vectors, vaccine carriers, and adjuvants [25]), antibody–drug conjugates (ADC), polymer therapeutics (polymer–protein conjugates or chemically modified proteins [26]), and liposomes, in line with some of the terms used in the JRC technical report [21]. Two “nanobodies” were in addition declared in two CTs and were included in a standalone classification, even if they are single-domain antibody fragments and nanoscale dimensions were not confirmed in the dossier. In Figure 3, we are reporting the categories attributable to nanomedicines identified across the 3 years investigated.

**Figure 4:**
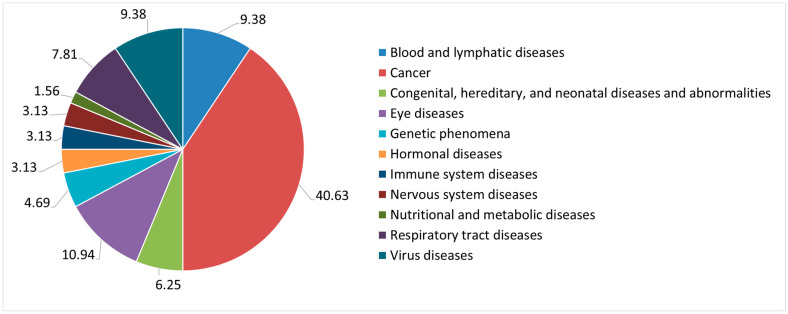
Distribution of therapeutic areas of CTs, including a nanomedicine tested as an IMP, assessed and authorized from 2018 to 2020.

Monoclonal antibodies, fusion proteins, and other recombinant products were not included in the scope because they are considered single biological molecules, even if their structural complexity is acknowledged. The same rationale applies for the three recombinant proteins that were not included, although dimensions in the nanoscale were confirmed by dynamic light scattering. CAR-T cells [27], and any other kind of cell therapy, were also not considered because their size was out of the nanoscale range. Although characterization data were provided for all IMPs, the nanoscale dimension has only been confirmed for 14 (21.88%) IMPs, while for the remaining 50 (78.12%) IMPs, the confirmation of the average size could not be retrieved. Nanocarriers, polymer therapeutics, antibody–drug conjugates, and nanobodies had already been retrieved in the analysis of the 2018 database, while in the analysis of the CTs authorized in the years 2019 and 2020, we also found IMPs that may be classified as liposomes. Liposomes are versatile drug delivery systems, developed for various routes of administration, that have the advantage of protecting the active substance enclosed in the vesicles, prolonging its half-life in the bloodstream and enhancing bioavailability. Several liposomal products have been authorized over the course of the last two decades by the European Medicines Agency (EMA) and the Food and Drug Administration (FDA) [28]. The distribution reported in Figure 4 shows that the vast majority of nanomedicines is tested in the therapeutic area of cancer, followed by eye disease, blood and lymphatic disease, virus disease, and respiratory tract disease therapeutic areas.

### 3.2. Quality Issues

Quality issues were detected in 51 (79.69%) out of the 64 authorized CTs. The number of quality issues identified from 2018 to 2020 and their classification, according to the current applicable guidelines on the requirements concerning IMPs in CTs [29,30], are reported in Table A3.

Globally, 822 quality issues were detected, with an average of 16.12 issues per CT, considering only those with objections. In the enumeration of issues, we included requests to update documents, data and information, clarifications, conditions, and recommendations on quality aspects of the application. All categories are impacted, with the only exception being the nomenclature of the drug substance.

**Figure 5:**
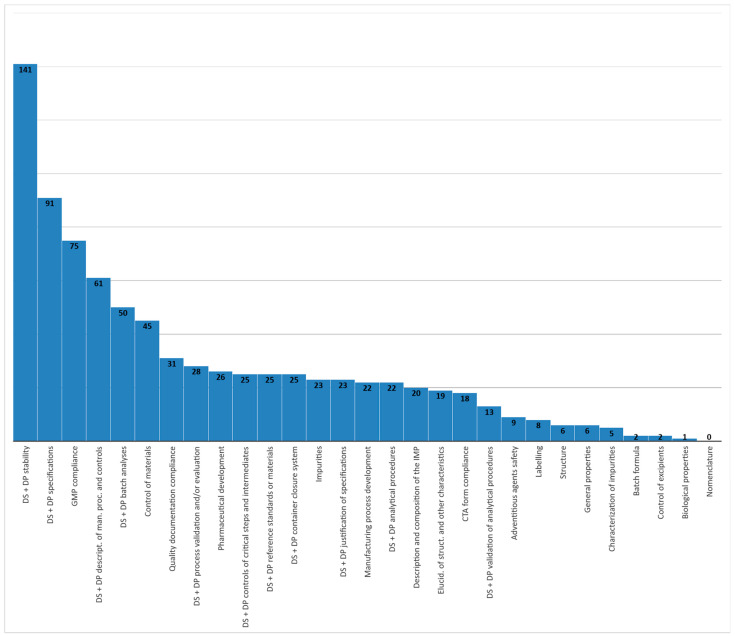
Number of quality issues combining drug substance (DS) and drug product (DP) classification label in CTs involving nanomedicines, assessed and authorized from 2018 to 2020.

The greatest number of issues detected concerns stability, both of the drug substance and of the drug product, representing cumulatively 17.15% of the overall number of issues. Specifications is the second area with regard to the number of issues detected, representing 11.07% of all issues. If we add issues regarding GMP compliance, description of the manufacturing process and process control, batch analyses, and control of materials, these first six classification labels together represent 56.33% of all issues. The subsequent types of issues impacting the quality profile in terms of numerical relevance are: quality documentation compliance, process validation and/or evaluation, pharmaceutical development, controls of critical steps and intermediates, reference standards or materials, container closure system, and impurities. Figure 5 provides details of the number of issues detected for each classification label.

#### 3.2.1. Stability

Considering the manufacturing date of batches reported in the IMPD, or the stability study start date, a request to provide updated stability data is often needed, whenever updated stability data should have been available but are not provided by the sponsor. In many other cases, stability data provided in the IMPD are limited and do not support the proposed retest or shelf life, particularly when an extrapolation is adopted. Other frequent requests are to investigate accelerated or stress conditions in order to identify potential degradation pathways or to justify out of specifications registered under normal conditions. It is also noted that a summary of results (including the description of the conditions tested, methods, and acceptance criteria) of the in-use stability and compatibility studies, which should support the product quality during clinical use, is not always provided, or, when it is provided, microbial parameters are sometimes missing. Another frequent issue is that product-related impurities are not tested at release and in stability studies. Concerning biological IMPs, the control of purity is mandatory and should be included in the release and stability testing. Subvisible particles is instead a test required by *Ph. Eur.* for parenteral preparations and should be controlled in the drug product both at release and during stability.

#### 3.2.2. Specifications

Drug substance and drug product batches intended to be used in CTs should be controlled with proper specifications and relevant tests, specifying the acceptance criteria. Depending on the type of drug substance or formulation of the drug product, a varied number of issues were detected. As an example, we report a non-exhaustive list of some of the significant criticalities encountered: solvents used in the last step of the synthesis (e.g., dichloromethane, heptane, and THF) not adequately controlled or amounts of these residual solvents not within ICH guideline limits; in parenteral preparations, subvisible particles or extractable volume not controlled; in suspensions or powders for injection, the uniformity of dosage units not controlled; for drug–device combinations, such as prefilled syringes, break loose and glide force, critical test parameters related to the functionality of the syringe, are not included in the specifications; the content of polysorbates, which are key excipients in biotechnological IMPs, not tested at release or during stability; for gene therapy products, transgene expression, vector aggregates, and genomic integrity of the vector not controlled at release by suitable methods.

#### 3.2.3. GMP Compliance

Manufacturing licenses (MIA), GMP certificates, and qualified person’s (QP) declaration of equivalence to EU GMP for IMPs manufactured in third countries (QP declaration) are frequently objects of non-compliance issues. Submitted MIAs sometimes do not cover IMPs but only authorized medicinal products. However, most of the issues are related to missing evidence that a manufacturer involved in the CT has a proper authorization to carry out the specific activities stated in the IMPD, such as batch certification of imported sterile biological medicinal products, quality control testing, primary or secondary packaging, and, in a few cases, also manufacturing activities. Concerning the QP declaration, the document is often requested to be updated because the list of EEA manufacturers reported therein is not matching the information provided in the IMPD or, less frequently, because the date of the audit to verify the EU GMP equivalence of the manufacturing sites is not recent.

#### 3.2.4. Description of the Manufacturing Process and Process Controls

Issues are generally related to the lack of details or information on the manufacturing process and on its control, and these may vary according to the specificity of the process. However, the most critical findings are noted on aseptic and sterilizing processes: the type and number of filters used per batch are not specified, data are not provided to demonstrate the compatibility with the filtered solution, and justification is not provided when the filter integrity test is conducted only after use. In addition, for the production of sterile medicinal products, it is necessary to provide information on the control of critical steps, such as the controls conducted to ascertain the efficiency of the production line in asepsis (e.g., media fill). Additional recurrent issues are noted when process intermediates are involved, and hold times and storage conditions are not justified and supported by data. In addition, when reprocessing is involved, it is not specified for which steps it is envisaged and in which cases. In this context, it is important to highlight that reprocessing could only be considered in exceptional circumstances, restricting these situations for biological products to certain re-filtration and re-concentration steps only, following a technical or mechanical failure of the equipment.

#### 3.2.5. Batch Analyses

The most recurrent issue is the submission of data that are not recent or are incomplete (e.g., missing manufacturing date or manufacturing site). In addition, data of clinical batches are sometimes not provided. Batches considered representative of the ones to be used in the CT, including batches of all relevant manufacturing processes and manufacturing sites, should be provided. Certificates of analysis (CoAs) for batches intended for use in the CT are often requested.

#### 3.2.6. Control of Materials

It is often necessary to request in-house specifications, including test methods and acceptance criteria, for non-compendial materials. Little information is usually submitted on the quality control of non-compendial raw materials, and representative CoAs, including specifications from suppliers and/or the in-house specifications for any testing performed on each non-compendial chemical raw materials, have to be requested. Sometimes, specifications provided do not include all the necessary controls, such as residual solvents or heavy metals. In general, a brief summary of the control of any critical attribute should be provided (e.g., if control is required to limit an impurity in the drug substance, or chiral control, metal catalyst control, or control of a precursor to a potential genotoxic impurity). In addition, the information provided should be representative of the process employed for the manufacture of the clinical supplies intended to be used in the CT.

#### 3.2.7. Quality Documentation Compliance

Most of the issues emerged from the need to update an IMPD that was not complete with all the required information and data. When updated documents are provided, substantial modifications should be listed and an updated version of the documents including track changes, as well as a clean version of the same documents, are expected to be submitted. An IMPD that was not representative of the IMP was rarely presented; more often instead, a cross-reference letter to an authorized version of the IMPD was not applicable because a more recent version had been authorized by the NCA or because the formulation of the referenced drug product was not deemed representative.

#### 3.2.8. Process Validation and/or Evaluation

Issues concerned missing information in the IMPD, able to confirm that process validation was performed. Usually, data should not be required during the development phases, except for non-standard sterilization processes. In particular, although the manufacturing process may be at an early stage of development, for sterile, aseptic manufacturing and lyophilization, the state of the validation should be briefly described, as well as the in-process controls applied. In this regard, it should be taken into consideration that the validation of sterilizing processes should be of the same standard as for products authorized for marketing. Therefore, justification of missing validation of sterilizing processes is requested, e.g., in the case of a sterilizing filtration. Clarifications are also usually requested on how the bioburden control is performed prior to the sterilizing filtration. When batch formula may vary according to clinical needs, information should be provided on the batch size that has been validated by media fill.

#### 3.2.9. Pharmaceutical Development

Development is still undergoing during CTs, and additional information is progressively collected on the manufacturing process, which is refined and optimized in due course. However, changes that occurred in the drug product manufacturing process during development are not always reported. When several process changes have been introduced (manufacturing scale, filter size, analytical methods for drug substance quantification, etc.) that could impact relevant quality attributes of the drug product, such as purity, the sponsor should support these changes with a comparability exercise, unless properly justified. The sponsor should provide a summary of the compatibility studies (including the description of the conditions tested, methods, acceptance criteria, and results) to support the product quality during clinical use. Discussion on leachable/extractable studies is expected for Phase III CTs.

#### 3.2.10. Controls of Critical Steps and Intermediates

If hold times are foreseen in the manufacturing process, the maximum hold time should be indicated for each step and a summary of data supporting each hold time should be provided. Hold times and storage conditions for intermediates should be justified and supported by data. Critical process parameters should be identified and stated (e.g., bioburden prior to sterile filtration, filters integrity, and fill weight) and, depending on the stage of development (e.g., Phase III), at least preliminary acceptance criteria should be established. The maximum acceptable bioburden prior to sterile filtration should be reported, and test volumes of less than 100 mL should be justified.

#### 3.2.11. Reference Standards or Materials

A reference standard should be established to ensure consistency between batches and comparability after process changes have been introduced. Adequate information on the reference standard and on its re-qualification is sometimes missing, and a request to clarify storage conditions and tests performed to confirm its stability over time is sometimes needed.

#### 3.2.12. Container Closure System

Analytical procedures used to control the container closure and any validations should be specified if the methods are not compendial. Specifications for all the container closure components are usually not provided. Release specifications for the packaging material is sometimes requested and so are data on compatibility between the solution and the stopper. The quality standard of the stopper should be indicated. In case non-compendial materials are used, description and specifications should be provided.

#### 3.2.13. Impurities

It is acknowledged that in the course of the development phase of an IMP, more in-depth knowledge is acquired and that at an early stage, less information may be available. However, the control of the impurity profile should always be present and upper limits, taking safety considerations into account, should be set; limits may then be reviewed and adjusted during development. Quantitative information on impurities should be provided, including the maximum amount for the highest clinical dose. Sometimes, not all types of impurities are taken into consideration (e.g., organic, inorganic, process-related, product-related, residual solvent, starting material, and potential mutagenic impurities). An inadequate control strategy is also a recurrent issue, e.g., not providing a justification based on a risk assessment for the proposed acceptance criteria. Data on the clearance of impurities and a safety assessment of the maximum amount per maximum dose in worst-case conditions are often requested and should be provided. Characterization of impurities was often found to be missing; at least the chemical structure should be provided in order to verify a potential toxicity profile. In connection with the setting and justification of specifications, particular focus on the limits set for process-related impurities and contaminants (e.g., bioburden and endotoxin) is needed. Impurities that are above the qualification threshold should be properly qualified by toxicological studies. Where a class 1 solvent might be present in another solvent (e.g., toluene and methanol containing benzene), a routine test for this class 1 solvent, on a suitable intermediate or on the final active substance, is required, unless an appropriate justification for residual levels is provided.

## 4. Discussion

This research is taking into account CTs authorized over a period of 3 years in Italy. For the 2018, a previous analysis [23] was conducted on the use of surfactants, nanomedicines, and nanocarriers in the context of CTs. We are now also investigating the quality issues and are adding those data to the databases of the following 2 years, 2019 and 2020, to provide a picture of the nanomedicine-related IMPs involved in CTs and the extent and type of quality issues detected across 3 years of cumulative activity at the CTO. CTs that were submitted to the CTO from 2018 to 2020 were quantified to be >22% of all those available in the EudraCT [31] system and therefore submitted in the EU in those years [22]. This pool of data can therefore represent a first estimate, even if partial, of the status in the overall EU.

Documentation submitted by the sponsors and information provided as structured data in the CTA form are not of help with the immediate identification of the presence of nanostructures tested as IMPs. In a few cases, a nanostructure is declared in the IMPD, but even in these cases, limited or no information is provided on dimension characterization. The nanoscale dimension has only been confirmed for 21.88% of the nanomedicine-related IMPs assessed. This issue is leading to a potential non-standardization in the characterization of nanotherapeutics. The CTO reacted by updating the draft submission cover letter [32] requesting sponsors to acknowledge if the CT involves the use of systems specially designed for clinical applications with at least one component in the nanometer scale from which specific and defined properties and characteristics derive, such as nanomedicines (nanocrystals, therapeutic polymers, albumin-bound nanoparticles, etc.), nanocarriers (e.g., liposomes, niosomes, nanoemulsions, micelles, and self-nanoemulsifying drug delivery systems (SNEDDs)), or nanodevices. Should a nanostructure be present, and should related information not be available within the IMPD, the sponsors are required to prepare an assessment of the benefit–risk associated with the nanomedicine, nanocarrier, or nanodevice to confirm the dimensions and to present a discussion of the nanotechnology used of the properties that may influence the kinetics and in vivo distribution, including a description of the analytical methods used for the characterization. These may include dynamic light scattering for hydrodynamic radius or polydispersion measurements; electron microscopy for morphology, purity, or core size; zeta potential, etc. This is a key point that should be taken up by the regulatory network to streamline and standardize the submission and assessment process of CTs involving nanotechnology, capitalizing on the sharing of best practices across NCAs.

In terms of nanomedicines’ classification, results show that only a few categories were detected, as reported in Figure 3, and that these do not include any non-ionic surfactant-based nanocarriers (e.g., micelles, nanoemulsions, and niosomes) or more complex structures or nanodevices. Reasons cannot be identified from the data in our possession, but we can easily imagine that some types of nanomedicines and/or formulations in the nanoscale may be more complex, facing additional difficulties. During the development phase, physicochemical properties and biological functions should be elucidated and adequate analytical methods and techniques should be implemented in order to define the safety and efficacy profile of the product under investigation in CTs. During the manufacturing scale-up phase, producibility and costs are crucial. These are other key points to address, as far as possible, also from a regulatory standpoint, by envisaging dedicated support to the development of specific kinds of nanomedicines that are acknowledged to require additional efforts. An expert working group on nanomedicines, a number of specific guidelines, and an innovation task force have been set by the EMA in the EU. However, additional approaches in a clinical trial setting could take the form of a detailed, standardized guideline for the submission of nanomedicines in CTs or could foresee additional opportunities for a structured and accessible early interaction between the regulatory framework and those stakeholders in or owners of innovative nanotechnology, such as researchers and academia, medium-sized enterprises (SMEs) or start-ups, and pharmaceutical companies that are bringing along with innovation potential new scientific and regulatory challenges not yet coded in any already known clinical trial framework (nanopharmaceuticals, complex trials, decentralized CTs, use of machine learning or artificial intelligence in CTs, big data and real-world evidence for regulatory decision purposes in CTs, etc.). In our analysis, CTs including an IMP that may be classified as a nanomedicine were distributed across 11 therapeutic areas: more than 1/3 (40.63%) were tested in cancer therapy, and the other most impacted therapeutic areas were eye disease (10.94%), blood and lymphatic disease (9.38%), virus disease (9.38%), and respiratory tract disease (7.81%).

Quality issues were detected in 51 (79.69%) out of 64 CTs. The average is 16.12 issues per CT, considering only those with objections. This result denotes that, in general, more attention needs to be paid to compiling quality data for IMPs in CTs. All sections of the IMPD, with the exception of nomenclature, were impacted by quality issues. Therefore, it is evident that additional efforts should be pursued by sponsors when preparing quality documentation. As an example, the sponsors could contract a specialized company that may aid in the elaboration of the dossier or that may perform an evaluation of the documentation before its submission to the regulatory agencies. Considering both the drug substance and the drug product sections of the IMPDs, most of the quality issues were retrieved in the areas of stability, which alone accounts for 17.15% of all issues; specifications (11.07%), GMP compliance (9.12%), and the description of the manufacturing processes and processes control (7.42%) are the three other labels with the greatest number of issues detected. Batch analyses and control of materials slightly differ from each other, accounting, respectively, for 6.08% and 5.47% of the issues. Then follows a cluster of labels with a lower impact in terms of the number of issues detected but significant because it covers a large area of parameters and characteristics impacting the quality profile of the IMP: quality documentation compliance, process validation and/or evaluation, pharmaceutical development, controls of critical steps and intermediates, reference standards or materials, container closure system, and impurities.

CTs are regulated by strict standards to guarantee the rights, safety, and well-being of subjects and the quality and integrity of data, and it is necessary to have the required know-how to be able to test an IMP, resources, and regulatory knowledge. However, even when all these requirements are accomplished, further efforts may be needed to ensure full compliance during the assessment process of a CT, especially for specific types of innovative products. However, a re-evaluation of the regulatory approach to innovation in the health sector, with particular reference to the use of nanotechnology in CTs, should be envisaged by the regulatory network, with the highest priority, in order to support the translation of innovation in a safe but also effective and faster way. Training programs, development of dedicated guidelines, an earlier confrontation with small realities that generate innovation, researchers and academia, dedicated funding for SMEs, and non-commercial (academic) sponsors are just a few examples of potential interventions that the regulatory network in collaboration with all the stakeholders could and should consider in the interests of public health protection and support of technological innovation.

## 5. Conclusions

A critical analysis on the quality documentation and information provided by sponsors, along with the submission of CTs, authorized at the CTO from 2018 to 2020, shows that only 3.17% of the authorized CTs are impacted by the use of nanomedicines. This confirms that nanotechnology innovation does not progress as fast as standard formulations when it comes to the clinical development of an IMP. The categories detected were: nanobody (3.13%); liposomes (7.81%), tested in five CTs; polymer therapeutics (29.69%); antibody–drug conjugates (28.13%); and nanocarriers (31.25%). Nanocarriers mainly include viral vectors; there is no evidence of the use of other structured delivery systems, such as non-ionic surfactant-based nanocarriers. Even if CTs are spread across 11 therapeutic areas, more than 1/3 (40.63%) of IMPs that may be classified as nanomedicines are tested in cancer therapy, followed by other therapeutic areas, such as eye disease (10.94%), blood and lymphatic disease (9.38%), virus disease (9.38%), and respiratory tract disease (7.81%). Almost all (95.31%) CTs have a commercial nature, and this reflects how difficult it may be to translate nanotechnology innovation into clinical development in the absence of adequate funding, know-how, resources, and regulatory expertise. This is a critical point that should be tackled, envisaging additional strategies to provide at least regulatory support to those academia and SMEs driving research in the nanotechnology innovation field. The use of systems appropriately designed for clinical applications with at least one component in the nanometric scale, from which specific and definite properties and characteristics may derive, is not properly coded as structured data in the CTA form, and it is not explicitly reported by the sponsors in the quality information of the CMC section of the IMPDs. As a consequence, their characterization may not be always standardized. In addition, when a nanostructure is mentioned, no adequate characterization in terms of dimension confirmation could be retrieved in the majority of cases. This is another crucial regulatory point to be addressed, suggesting the need for a dedicated guideline on the assessment of nanotechnology-enabled IMPs in CTs.

The quality issues detected during the assessment of IMPs that may be classified as nanomedicines are shared, discussing for the first time the results of the assessment reports elaborated by the quality assessors at the CTO. For this research, we focused on CTs assessed and authorized from 2018 to 2020, which included a nanomedicine tested as an IMP, as explained above. Results confirm that the highest quality standards are guaranteed by the assessment process and are ensured before the authorization of a CT. Quality issues were detected for almost all sections of the IMPDs submitted, outlining that, in general, the quality of applications and related quality documentation should definitely be improved. In quantitative terms, considering both the drug substance and the drug product, the definition of the stability profile is the criticality with the greatest impact on the clinical development process of IMPs involving nanostructures. Other areas of major impact, both for the substance and the product, are specifications, compliance with GMPs, description of the manufacturing processes and process controls, batch analysis, and control of materials during manufacture. The compliance of quality documentation, process validation and/or evaluation, the pharmaceutical development, the controls of critical steps and intermediates, the reference standards or materials, the container closure system, and the impurity profile during the characterization of the active substance are additional sectors to which more attention needs to be paid. Findings provide valuable information to sponsors of CTs and developers of nanomedicines to focus on those areas of potential difficulty. Results should be capitalized on, leading to the development of a regulatory approach to innovation that takes into account the criticalities that emerge in due course of the investigations and the scientific evidence, suggesting improvements to the translation of innovation, continuing to guarantee the highest level of safety but at the same time supporting a more rapid, smooth, and effective application in a CT setting.

## Figures and Tables

**Table 1:** CTs submitted, authorized, and within the scope of this research.

Year	CTs Submitted	CTs Authorized	CTs in Scope
2018	716	666	**433**
2019	722	672	**449**
2020	815	683	**359**

## Data Availability

Additional information on the data presented in this study is available on request from the corresponding author. The data are not publicly available due to the protection of commercially confidential information.

## Appendix A

**Table A1:** Nanomedicines detected in CTs authorized in 2019.

Code	Description	Nanomedicine-Related Term	Analytical Method Confirming Nanoscale Dimension	Pharmaceutical Form	Study Phase	Therapeutic Area	Active Substance of Chemical Origin	Active Substance of Biological/Biotechnological Origin	Gene Therapy Medicinal Product
N23	RNA–peptide	Polymer	Yes	Solution for injection	I	Cancer	No	Yes	No
	complex	therapeutic							
N24	Recombinant type 5 adenovirus vector	Nanocarrier	Yes	Concentrate for solution for infusion ev	III	Cancer	No	No	Yes
N25	Pegylated protein	Polymer	No	Solution for injection	I/II	Cancer	No	Yes	No
		therapeutic							
N17	Pegylated peptide	Polymer	No	Solution for infusion	III	Blood and lymphatic	Yes	No	No
		therapeutic				diseases			
N26	Recombinant	Nanocarrier	No	Solution for infusion ev	III	Blood and lymphatic	No	No	Yes
	adeno-associated virus					diseases			
	vector serotype 5								
N27	Monoclonal antibody conjugated to a fluorochrome through a linker	Antibody–drug conjugate (ADC)	No	Concentrate for solution for injection	III	Cancer	No	Yes	No
N28	Monoclonal antibody conjugated to a cytotoxic agent through a linker	Antibody–	No	Powder for concentrate for solution for infusion	III	Cancer	No	Yes	No
		drug							
		conjugate							
		(ADC)							
N29	Recombinant protein attached to an albumin binding moiety	Nanocarrier	No	Solution for injection	III	Hormonal diseases	No	Yes	No
N30	Recombinant adenovirus serotype 155 viral vector	Nanocarrier	No	Suspension for injection	I	Respiratory tract diseases	No	Yes	No
N17	Pegylated peptide	Polymer	No	Solution for injection	III	Eye diseases	Yes	No	No
		therapeutic							
N17	Pegylated peptide	Polymer	No	Solution for injection	III	Eye diseases	Yes	No	No
		therapeutic							
N7	Pegylated enzyme	Polymer	No	Concentrate for solution for infusion	III	Body processes—genetic phenomena	No	Yes	No
		therapeutic							
N31	Recombinant adeno-associated viral vector	Nanocarrier	Yes	Concentrate for solution for infusion	I/II	Congenital, hereditary, and neonatal diseases and abnormalities	No	No	Yes
N32	Liposomal adjuvant	Liposome	Yes	Powder for solution for injection	II	Respiratory tract diseases	No	Yes	No
N33	Beads coated with the active ingredient	Nanocarrier	No	Soft capsule	II	Immune system diseases	No	Yes	No
N34	Pegylated recombinant protein	Polymer	No	Powder for solution for injection	II	Cancer	No	Yes	No
		therapeutic							
N35	Recombinant adeno virus vector serotype 26	Nanocarrier	Yes	Solution for injection	III	Virus diseases	No	Yes	No
N36	Recombinant adeno-associated virus vector serotype 2	Nanocarrier	No	Solution for injection	I/II	Blood and lymphatic diseases	No	No	Yes
N37	Pegylated peptide	Polymer	No	Solution for injection	II	Hormonal diseases	Yes	No	No
		therapeutic							
N38	Pegylated enzyme	Polymer	No	Concentrate for solution for infusion ev	III	Congenital, hereditary, and neonatal diseases and abnormalities	No	Yes	No
		Therapeutic							
N39	Liposome-based adjuvant	Liposome	Yes	Powder for solution for injection	II	Virus diseases	No	Yes	No
N28	Monoclonal antibody conjugated to a cytotoxic agent through a linker	Antibody–	No	Powder for concentrate for solution for infusion	III	Cancer	No	Yes	No
		drug							
		conjugate							
		(ADC)							
N28	Monoclonal antibody conjugated to a cytotoxic agent through a linker	Antibody–	No	Powder for concentrate for solution for infusion	III	Cancer	No	Yes	No
		drug							
		conjugate							
		(ADC)							

**Table A2:** Nanomedicines detected in CTs authorized in 2020.

Code	Description	Nanomedicine-Related Term	Analytical Method Confirming Nanoscale Dimension	Pharmaceutical Form	Study Phase	Therapeutic Area	Active Substance of Chemical Origin	Active Substance of Biological/Biotechnological Origin	Gene Therapy Medicinal Product
N40	Monoclonal antibody	Antibody–drug conjugate (ADC)	No	Powder for solution for injection	II	Cancer	No	Yes	No
	conjugated to a								
	cytotoxic agent through								
	a linker								
N41	Plasmid vector	Nanocarrier	No	Solution for injection	II	Cancer	No	Yes	No
N42	Antibody conjugated with a biopolymer	Polymer therapeutic	No	Solution for injection	II	Eye diseases	No	Yes	No
N43	Adeno-associated virus serotype 9 vector	Nanocarrier	No	Concentrate for solution for infusion ev	III	Nervous system diseases	No	No	Yes
N44	Monoclonal antibody	Antibody–drug conjugate (ADC)	Yes	Concentrate for solution for infusion	III	Cancer	No	Yes	No
	conjugated to a								
	cytotoxic agent through								
	a linker								
N45	Monoclonal antibody	Antibody–drug conjugate (ADC)	No	Powder for solution for infusion	I	Cancer	No	Yes	No
	conjugated to a								
	cytotoxic agent through								
	a linker								
N46	Monoclonal antibody conjugated to a cytotoxic agent through a linker	Antibody–drug conjugate (ADC)	No	Concentrate for solution for infusion	III	Cancer	No	Yes	No
N7	Pegylated enzyme	Polymer	No	Concentrate for solution for infusion	III	Body processes—genetic phenomena	No	Yes	No
		therapeutic							
N47	Pegylated oligonucleotide	Polymer therapeutic	No	Solution for injection	III	Eye diseases	Yes	No	No
N48	Adenoviral vector	Nanocarrier	No	Suspension for injection	I	Virus diseases	Yes	Yes	No
N34	Pegylated recombinant protein	Polymer therapeutic	No	Powder for solution for injection	III	Cancer	No	Yes	No
N49	Trivalent nanobody	Nanobody	No	Solution for infusion ev	II	Cancer	No	Yes	No
N50	Pegylated monoclonal antibody	Polymer therapeutic	No	Powder for solution for infusion ev	III	Immune system processes	No	Yes	No
N51	Liposomal formulation	Liposome	Yes	Powder for nebulization solution	II	Virus diseases	Yes	No	No
N52	Monoclonal antibody conjugated to a cytotoxic agent through a linker	Antibody–drug conjugate (ADC)	No	Powder for solution for infusion ev	II	Cancer	No	Yes	No
N53	Liposome suspension	Liposome	No	Inhalation suspension	III	Respiratory tract diseases	Yes	No	No
N54	Adenoviral vector	Nanocarrier	Yes	Solution for injection	III	Respiratory tract diseases	No	Yes	No
N42	Antibody conjugated with a biopolymer	Polymer therapeutic	No	Solution for injection	III	Eye diseases	No	Yes	No
N53	Liposome suspension	Liposome	No	Inhalation suspension	III	Respiratory tract diseases	Yes	No	No

**Table A3:** Number of quality issues and their classification for CTs assessed and authorized from 2018 to 2020.

Classification Label of Quality Issues		Totals Per Classification
CTA Form Compliance		18
Quality documentation compliance (IMPD, S-IMPD, SmPC, CE mark)		31
GMP compliance: information about all manufacturers involved (drug substance, drug product) and evidence of GMP (manufacturing licenses/GMP certificates, QP declarations, CEPs provided)		75
**Drug Substance (DS)**		
General information	Nomenclature	0
	Structure	6
	General properties	6
	Biological properties	1
Manufacture	Description of manufacturing process and process controls	42
	Control of materials	45
	Control of critical steps and intermediates	15
	Process validation and/or evaluation	6
	Manufacturing process development	22
Characterization	Elucidation of structure and other characteristics	19
	Impurities	23
Control of drug substance	Specifications	44
	Analytical procedures	13
	Validation of analytical procedures	9
	Batch analyses	33
	Justification of specification(s)	12
Reference standards or materials		20
Container closure system		9
Stability		57
**Drug Product (DP)**		
Description and composition of the investigational medicinal product		20
Pharmaceutical development		26
Manufacture	Batch formula	2
	Description of manufacturing process and process controls	19
	Controls of critical steps and intermediates	10
	Process validation and/or evaluation	22
Control of excipients		2
Control of drug product	Specifications	47
	Analytical procedures	9
	Validation of analytical procedures	4
	Batch analyses	17
	Characterization of impurities	5
	Justification of specification(s)	11
Reference standards or materials		5
Container closure system		16
Stability		84
Labeling		8
Adventitious agents’ safety		9
TOTAL		822

## References

[1] Samrot A.V., Sean T.C., Kudaiyappan T., Bisyarah U., Mirarmandi A., Faradjeva E., Abubakar A., Ali H.H., Angalene J.L.A., Kumar S.S. (2020). Production, characterization and application of nanocarriers made of polysaccharides, proteins, bio-polyesters and other biopolymers: A review. Int. J. Biol. Macromol..

[2] Aminu N., Bello I., Umar N.M., Tanko N., Aminu A., Audu M.M. (2020). The influence of nanoparticulate drug delivery systems in drug therapy. J. Drug Deliv. Sci. Technol..

[3] Patra J.K., Das G., Fraceto L.F., Campos E.V.R., del Pilar Rodriguez-Torres M., Acosta-Torres L.S., Diaz-Torres L.A., Grillo R., Swamy M.K., Sharma S. (2018). Nano based drug delivery systems: Recent developments and future prospects. J. Nanobiotechnol..

[4] Longo J.P.F., Muehlmann L.A., Calderón M., Stockmann C., Azevedo R.B. (2021). Editorial: Nanomedicine in Cancer Targeting and Therapy. Front. Oncol..

[5] Prasad M., Lambe U.P., Brar B., Shah I., Manimegalaj J., Ranjan K., Rao R., Kumar S., Mahant S., Khurana S.K. (2017). Nanotherapeutics: An insight into healthcare and multi-dimensional applications in medical sector of the modern world. Biomed. Pharmacother..

[6] Ventola C.L. (2017). Progress in Nanomedicine: Approved and Investigational Nanodrugs. Pharm. Ther..

[7] Farjadian F., Ghasemi A., Gohari O., Roointan A., Karimi M., Hamblin M.R. (2019). Nanopharmaceuticals and nanomedicines currently on the market: Challenges and opportunities. Nanomedicine.

[8] Sa-Nguanmoo N., Namdee K., Khongkow M., Ruktanonchai U., Zhao Y., Liang X.-J. (2021). Review: Development of SARS-CoV-2 immuno-enhanced COVID-19 vaccines with nano-platform. Nano Res..

[9] Longo J.P.F., Muehlmann L.A. (2021). How has nanomedical innovation contributed to the COVID-19 vaccine development?. Nanomedicine.

[10] Bhattacharjee S., Brayden D.J. (2021). Addressing the challenges to increase the efficiency of translating nanomedicine formulations to patients. Expert Opin. Drug Discov..

[11] Germain M., Caputo F., Metcalfe S., Tosi G., Spring K., Åslund A.K., Pottier A., Schiffelers R., Ceccaldi A., Schmid R. (2020). Delivering the power of nanomedicine to patients today. J. Control. Release.

[12] Đorđević S., Gonzalez M.M., Conejos-Sánchez I., Carreira B., Pozzi S., Acúrcio R.C., Satchi-Fainaro R., Florindo H.F., Vicent M.J. (2022). Current hurdles to the translation of nanomedicines from bench to the clinic. Drug Deliv. Transl. Res..

[13] Halwani A.A. (2022). Development of Pharmaceutical Nanomedicines: From the Bench to the Market. Pharmaceutics.

[14] Kapoor B., Gupta R., Gulati M., Singh S.K., Khursheed R., Gupta M. (2019). The Why, Where, Who, How, and What of the vesicular delivery systems. Adv. Colloid Interface Sci..

[15] European Commission (2010). EudraLex—Volume 10—Clinical Trials Guidelines. https://ec.europa.eu/health/documents/eudralex/vol-10_en.

[16] Directive 2001/20/EC of the European Parliament and of the Council of 4 April 2001 on the Approximation of the Laws, Regulations and Administrative Provisions of the Member States Relating to the Implementation of Good Clinical Practice in the Conduct of Clinical Trials on Medicinal Products for Human Use. https://eurlex.europa.eu/LexUriServ/LexUriServ.do?uri=OJ:L:2001:121:0034:0044:en:PDF.

[17] European Commission (2010). Annex 13 to Volume 4, EU Guidelines to Good Manufacturing Practice, Medicinal Products for Human and Veterinary Use. https://ec.europa.eu/health/sites/default/files/files/eudralex/vol-4/2009_06_annex13.pdf.

[18] Regulation (EU) No 536/2014 of the European Parliament and of the Council of 16 April 2014 on Clinical Trials on Medicinal Products for Human Use, and Repealing Directive 2001/20/EC. https://ec.europa.eu/health/sites/default/files/files/eudralex/vol-1/reg_2014_536/reg_2014_536_en.pdf.

[19] Italian Minister of Health Decree Dated 21 December 2007. https://www.gazzettaufficiale.it/atto/serie_generale/caricaDettaglioAtto/originario?atto.dataPubblicazioneGazzetta=2008-03-03&atto.codiceRedazionale=08A01360&elenco30giorni=false.

[20] AIFA (2022). Osservatorio Nazionale Sperimentazione Clinica. https://www.aifa.gov.it/osservatorio-nazionale-sperimentazione-clinica.

[21] Quirós Pesudo L., Balahur A., Gottardo S., Rasmussen K., Wagner G., Joanny G., Bremer-Hoffmann S. (2018). Mapping Nano-medicine Terminology in the Regulatory Landscape.

[22] AIFA (2022). Rapporto Sulla Sperimentazione Clinica dei Medicinali in Italia. https://www.aifa.gov.it/rapporto-sulla-sperimentazione-clinica-dei-medicinali-in-italia.

[23] Dri D.A., Marianecci C., Carafa M., Gaucci E., Gramaglia D. (2021). Surfactants, Nanomedicines and Nanocarriers: A Critical Evaluation on Clinical Trials. Pharmaceutics.

[24] European Commission Annex 1: Clinical trial Application Form Request for Authorisation of a Clinical Trial on a Medicinal Product for Human Use to the Competent Authorities and for Opinion of the Ethics Committees in the Community. https://ec.europa.eu/health/system/files/2019-11/application-form_en_0.pdf.

[25] Van Kan-Davelaar H.E., Van Hest J.C.M., Cornelissen J.J.L.M., Koay M.S.T. (2014). Using viruses as nanomedicines. J. Cereb. Blood Flow Metab..

[26] Choi Y.H., Han H.-K. (2019). Nanomedicines: Current status and future perspectives in aspect of drug delivery and pharmacokinetics. J. Pharm. Investig..

[27] Titov A., Zmievskaya E., Ganeeva I., Valiullina A., Petukhov A., Rakhmatullina A., Miftakhova R., Fainshtein M., Rizvanov A., Bulatov E. (2021). Adoptive Immunotherapy beyond CAR T-Cells. Cancers.

[28] Liu P., Chen G., Zhang J. (2022). A Review of Liposomes as a Drug Delivery System: Current Status of Approved Products, Regulatory Environments, and Future Perspectives. Molecules.

[29] European Medicines Agency (2022). Committee for Medicinal Products for Human Use (CHMP)-Guideline on the Requirements for the Chemical and Pharmaceutical Quality Documentation Concerning Investigational Medicinal Products in Clinical Trials. https://www.ema.europa.eu/en/documents/scientific-guideline/guideline-requirements-chemical-pharmaceutical-quality-documentation-concerning-investigational_en-1.pdf.

[30] European Medicines Agency (2022). Committee for Medicinal Products for Human Use (CHMP)-Guideline on the Requirements for Quality Documentation Concerning Biological Investigational Medicinal Products in Clinical Trials. https://www.ema.europa.eu/en/documents/scientific-guideline/guideline-requirements-quality-documentation-concerning-biological-investigational-medicinal_en-2.pdf.

[31] EudraCT. https://eudract.ema.europa.eu.

[32] AIFA (2019). Aggiornamento dei Modelli Delle Lettere di Ttrasmissione e Della Documentazione da Sottomettere per l’Autorizzazione di Sperimentazioni Cliniche e Relativi Emendamenti Sostanziali. https://www.aifa.gov.it/documents/20142/0/comunicazione_agg_mod_SC-ES_2019_08_01.pdf.

